# Functional Status, Daily Functional Changes, and Cognitive Function in Low-Income Older Adults

**DOI:** 10.1093/geroni/igaf122.3595

**Published:** 2025-12-31

**Authors:** Renata Komalasari, Ni Gusti Eka, Martina Pakpahan, Vivien Puspitasari, Peggy Tahulending, Rita Chang, Michael Dino, Ladda Thiamwong

**Affiliations:** Akademi Keperawatan Andalusia Jakarta, Jakarta, Jakarta Raya, Indonesia; Universitas Pelita Harapan, Universitas Pelita Harapan, Banten, Indonesia; Universitas Pelita Harapan, Tangerang, Banten, Indonesia; Universitas Pelita Harapan, Tangerang, Banten, Indonesia; Mayapada Hospital, Mayapada Hospital, Jakarta Raya, Indonesia; Western sydney university, Penrith, New South Wales, Australia; University of Central Florida, University of Central Florida, Florida, United States; University of Central Florida, Orlando, Florida, United States

## Abstract

Despite consistent evidence on the association between functional status and cognitive function, there is a lack of understanding on daily functional changes in community-dwelling older adults in Indonesia. We examined associations between functional status, healthy brain aging, and cognitive function among 150 adults aged ≥60 (60.7% female, Mage=65.54, SD ± 4.65) in low-income settings, Tangerang. The six-item Rowland Universal Dementia Assessment Scale (RUDAS-Ina) was used to assess cognitive function. Functional status was measured with the eight-item Lawton Instrumental Activities of Daily Living (IADL) Scale. The ten-domain Healthy Brain Aging form assessed functional changes in daily functioning within the past twelve months. Covariates were dementia severity rating scale and sociodemographic. We used generalized linear models with a Poisson distribution and a log link, which is robust to violations of normality in outcome measures. Findings showed functional status was significantly related to cognitive function (e1.079= 0.076, p = < 0.001), but functional changes were not, controlling for dementia severity rating scale and sociodemographic. For every one unit increase in IADL scores, the RUDAS scores increase 7.6%. Mann-Whitney U test was conducted to determine if there were differences in cognitive function. There was a significant difference between higher (RUDAS-Ina scores ≥23) (n = 121) and lower cognitive status group (RUDAS-Ina scores < 23) (n = 29): U = 2774.00, p <.001. Older adults who maintained independent functional performance tended to enjoy healthier cognitive function than their counterparts. Older adults should strive to maintain their independence in performing daily activities to promote healthy cognition, supporting more active aging.

